# Parathyroid Carcinoma Complicated by Parathyromatosis and Refractory Hypercalcemia

**DOI:** 10.7759/cureus.72584

**Published:** 2024-10-28

**Authors:** Yug Garg, Madhumati S Vaishnav, Nidhi Garg, Kavitha Muniraj, Sathyanarayana Srikanta

**Affiliations:** 1 Department of Endocrinology Diabetes Medicine, Samatvam Diabetes Endocrinology and Medical Center Samatvam: Science and Research for Human Welfare Trust, Bangalore, IND

**Keywords:** cinacalcet, denosumab, granulomatous lymphadenitis, hypercalcemic crisis, nutritional vitamin d deficiency, parathyroid carcinoma, parathyroidectomy, parathyromatosis, primary hyperparathyroidism, zoledronic acid

## Abstract

Parathyroid carcinoma (PC) is a rare malignancy. In January 2022, a 41-year-old woman presented with weight loss, proximal muscle weakness, and bone pain. She was diagnosed with severe hypercalcemia with serum calcium of 15.5 mg/dL (8.8-10.6 mg/dL). A biopsy of cervical lymphadenopathy revealed non-caseating granulomatous lymphadenitis. Primary hyperparathyroidism was subsequently confirmed with parathormone (PTH) exceeding 2,500 pg/mL (12-88 pg/mL), leading to left-superior parathyroidectomy and hemithyroidectomy. Histopathology suggested PC versus adenoma, with oxyphilic cells. Postoperatively, she manifested severe hypocalcemia and vitamin D deficiency alongside elevated PTH levels. In January 2023, she experienced a hypercalcemic crisis and developed a new right-sided neck mass. Wide excision revealed PC with parathyromatosis. By September 2023, another hypercalcemic crisis and new left cervical nodules necessitated further surgery, confirming PC deposits in the neck, though without lymph node metastases. Despite treatment with cinacalcet and zoledronic acid, her hypercalcemia persisted until denosumab produced a dramatic response (serum calcium dropping from 16.7 to 7.9 mg/dL; PTH 1,168 pg/mL). However, she remains at risk for progressive local disease and potential distant metastases.

## Introduction

Parathyroid carcinoma (PC) is a rare and aggressive endocrine malignancy, accounting for less than 1% of all cases of sporadic primary hyperparathyroidism. Despite its low prevalence, PC poses considerable challenges in both diagnosis and management due to its often indolent course, followed by progressive clinical deterioration. Patients with PC typically present with severe hypercalcemia, which is often refractory to standard medical management, making early detection crucial to improving patient outcomes. However, diagnosis is frequently delayed, as the clinical and biochemical features of PC overlap with benign causes of hyperparathyroidism, such as adenomas. This delay is further compounded by the fact that histopathological distinction between benign and malignant parathyroid lesions can be challenging, with many tumors exhibiting equivocal or non-specific features at initial presentation. Only in cases of local recurrence, distant metastasis, or invasive behavior do the true malignant nature of the tumor become apparent, making early-stage identification difficult for clinicians [1-6].

In understanding PC, it is important to appreciate the broader implications of its presentation, not only as a malignant disease but also as a contributor to severe metabolic disturbances, particularly hypercalcemia. Hypercalcemia associated with PC often requires aggressive therapeutic strategies and can become life-threatening if not promptly addressed. Additionally, the presence of parathyromatosis, a condition characterized by the seeding and growth of hyperfunctioning parathyroid tissue throughout the neck or mediastinum, further complicates the clinical picture. Parathyromatosis, often occurring after surgical manipulation, can lead to recurrent and refractory hypercalcemia, presenting ongoing management difficulties. Granulomatous lymphadenitis and severe nutritional vitamin D deficiency, as seen in this case, add further complexity to the diagnosis and treatment of this rare malignancy.

This case report highlights the intricate diagnostic and therapeutic challenges in managing PC, especially when complicated by parathyromatosis and refractory hypercalcemia. It underscores the critical importance of a multidisciplinary approach - encompassing endocrinologists, surgeons, pathologists, radiologists, and nuclear medicine specialists - to improve patient outcomes. Additionally, this case draws attention to the necessity of considering rare complications in patients with persistent hyperparathyroidism, even after surgical intervention. By sharing this unique clinical experience, we aim to contribute valuable insights to the existing literature and provide clinicians with practical guidance on managing such complex cases. Ultimately, a better understanding of the broader significance of PC and its complications will facilitate earlier diagnosis and more effective treatment strategies.

This article was previously presented as a meeting abstract at the 21st International Congress of Endocrinology, Dubai, March 1-3, 2024.

## Case presentation

**Figure 1:**
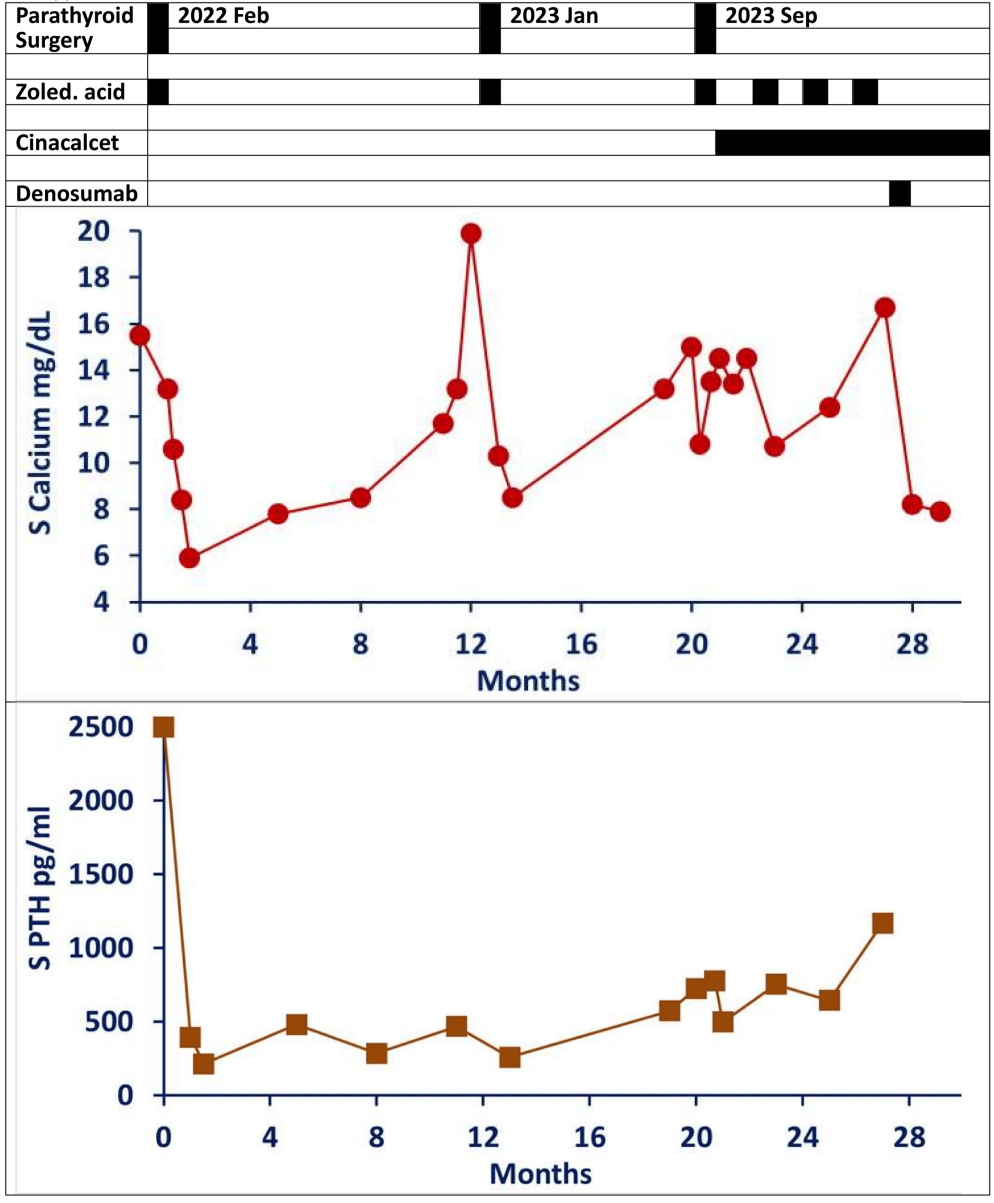
Serial clinical course, biochemical parameters, and management approaches Normal reference ranges: serum calcium mg/dL (mmol/L) = 8.8-10.6 (2.20-2.65); serum phosphorus mg/dL (mmol/L) = 2.4-5.1 (0.78-1.65); serum parathormone (PTH)/parathyroid hormone pg/mL (pmol/L) = 12-88 (1.27-9.33); serum 25-hydroxy vitamin D ng/mL (nmol/L) = >30 (>75); serum alkaline phosphatase U/L (μkat/L) = 45-129 (0.75-2.15); serum creatinine mg/dL (μmol/L) = 0.55-1.02 (48.6-90.2).

Clinical, biochemical, imaging, surgical, histopathological, and management details of this case are described below (Table 1, Figure 1 to Figure 5). Additional information can be found in Appendix A and Appendix B in the Appendices.

**Table 1:** Serial clinical course, biochemical parameters, and management approaches S Alk Phos = serum alkaline phosphatase; S Cal = serum calcium; S Crea = serum creatinine; S Phos = serum phosphorus; S PTH = serum parathyroid hormone; S 25 Vit D = serum 25-hydroxy vitamin D

Time	Clinical Features/Diagnosis	S Cal (mg/dL) (normal range: 8.8-10.6)	S Phos (mg/dL) (normal range: 2.4-5.1)	S PTH (pg/mL) (normal range: 12-88)	S 25 Vit D (ng/mL) (normal range: >30)	S Alk Phos (U/L) (normal range:45-129)	S Crea (mg/dL) (normal range: 0.55-1.02)	Medical/Surgical Management
2022								
January	Weight loss, proximal muscle weakness, hip and back pain, hypercalcemia, left cervical/supraclavicular lymphadenopathy	15.5	-	-	-	535	1.35	Inj zoledronic acid IV, lymph node excision
February	Hyperparathyroidism primary; left superior parathyroid adenoma versus carcinoma	13.2-10.6	-	>2,500	-	-	1.25	Left superior parathyroidectomy, left hemithyroidectomy
February	Hypocalcemia, vitamin D deficiency; hyperparathyroidism primary + secondary	8.4-5.9	-	394-213	3.0	-	1.00	Treatment: oral calcium carbonate and vitamin D (low dose)
June	Endocrinology consult, wheel chair bound, severe aches and pains, pseudofractures, renal calculi	7.8	2.20	480	-	743	0.79	Osteomalacia myopathy; treatment: vitamin D; active vitamin D
September	-	8.5	-	286	29.0	538	0.90	-
December	Hypercalcemia	11.7-13.2	2.69	467	33.6	297	0.76	-
2023								
January	Hypercalcemic crisis, swelling in right lower neck cystic solid; right parathyromatosis	19.9	-	-	-	-	-	Inj zoledronic acid IV 5 mg, wide excision of right neck mass/block dissection
February	-	10.3-8.5	1.82	258	38.8	316	0.70	-
August	Hypercalcemia	13.2	2.09	572	-	-	-	-
September	Hypercalcemic crisis; parathyroid carcinoma deposits in neck soft tissues - left side	15.0	2.26	725	36.0	370	0.86	Inj zoledronic acid IV 5 mg, left cervical neck dissection/lymph node excision
September	-	10.8	-	-	-	471	0.80	-
September	Hypercalcemia	13.5	1.90	778	-	-	-	Tab cinacalcet 60 mg BD
October	Hypercalcemia	14.5-13.4	2.00	498	-	-	-	-
November	Hypercalcemia	14.5	-	-	-	-	-	Inj zoledronic acid IV 5 mg
December	-	10.7	2.00	754	-	-	-	-
2024								
February	Hypercalcemia	12.4	2.16	644	24	-	-	Inj zoledronic acid IV 5 mg
April	Hypercalcemic crisis	16.7	4.00	1168	46.5	-	1.4	Inj zoledronic acid IV 5 mg
May	Dramatic response	8.2	1.10	-	39	>1,000	0.73	Inj denosumab subcutaneous (SC) 120 mg
June	Clinical improvement	7.9	1.17	-	-	-	0.58	-

**Figure 2:**
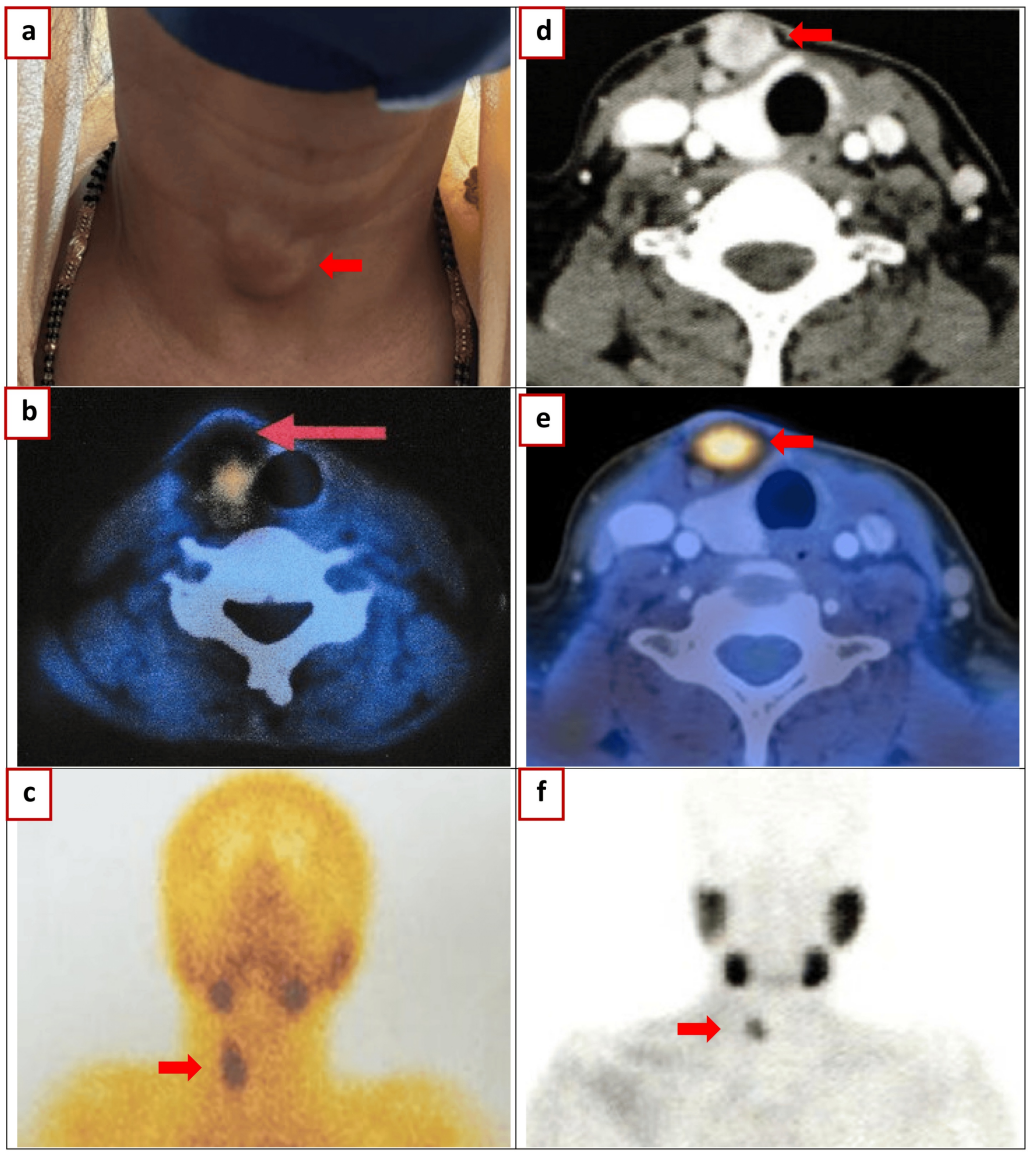
Nuclear medicine imaging (a-c) 2023 January: Technetium (Tc) 99m sestamibi single photon emission computed tomography (SPECT) CT scan: Subcutaneous cystic lesion anterior to the right lobe of the thyroid. No other evidence of abnormal focal increased MIBI uptake in the neck or mediastinum. (d-f) 2023 January: 11-Choline PET-CT: Subcutaneous nodule (17 × 15 mm) in right lower anterior neck standardized uptake value (SUV): 5.8. Left lower cervical and supraclavicular lymph nodes SUV: 2.2 (largest: 11 × 9 mm).

**Figure 3:**
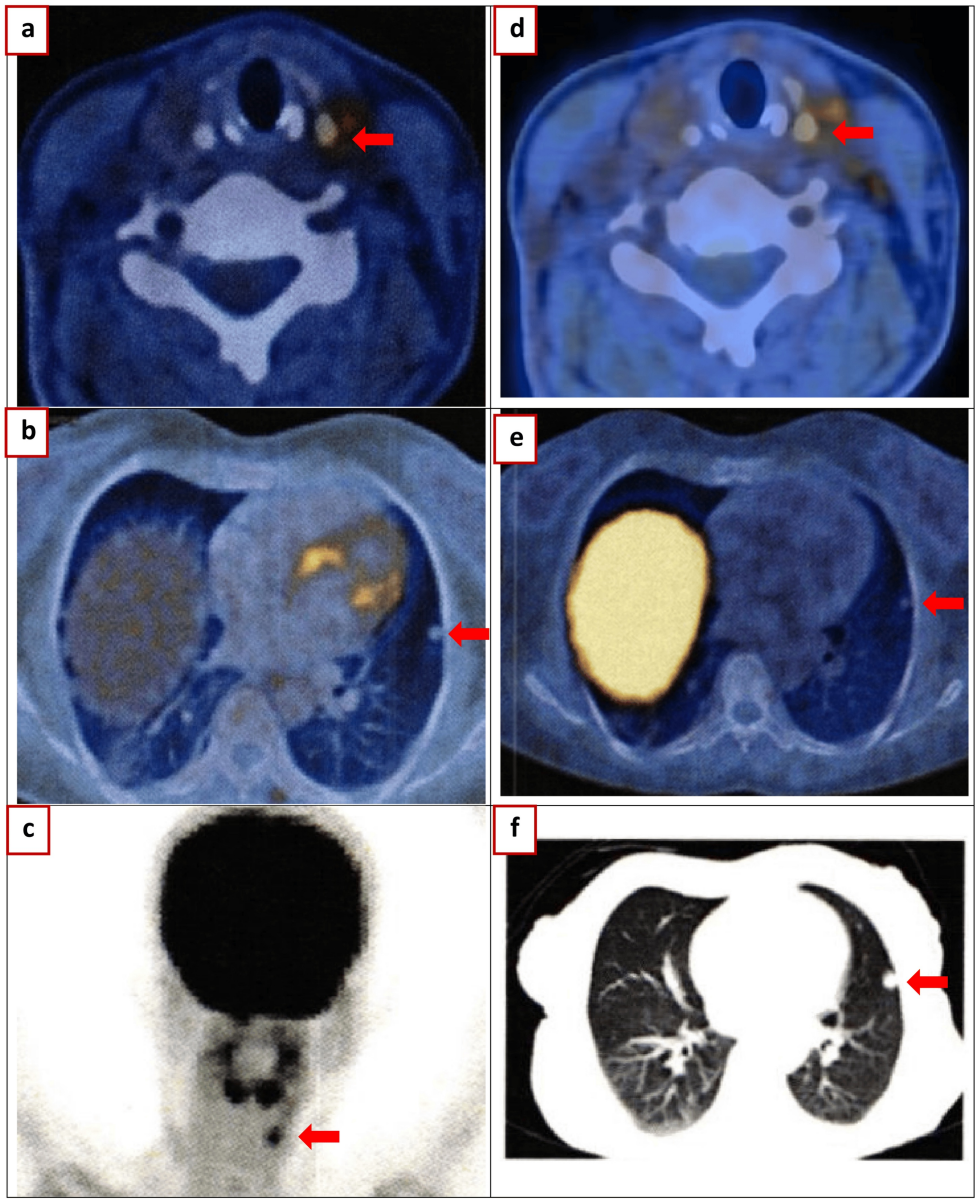
Nuclear medicine imaging (a-c) 2023 August: 18 F-fluorodeoxyglucose (FDG) PET/CT: “New” nodule/lymph node in the left mid jugular region standardized uptake value (SUV): 4.8 (8 × 6 mm). Left lower cervical and supraclavicular lymph nodes SUV: 1.9 (largest 11 × 9 mm). Left lung nodules SUV: 1.6 (7.5 mm). (d,e) 2023 August: Ga68 Dotanoc PET-CT: “New” nodule/lymph node in the left mid jugular region SUV: 3.2 (8 × 6 mm). Left lower cervical and supraclavicular lymph nodes SUV: 1.6 (largest 11 × 9 mm). Left lung nodules SUV: 1.6 (7.5 mm). (f) 2024 May: Chest CT scan showing metastatic left lower lobe lung nodule.

**Figure 4:**
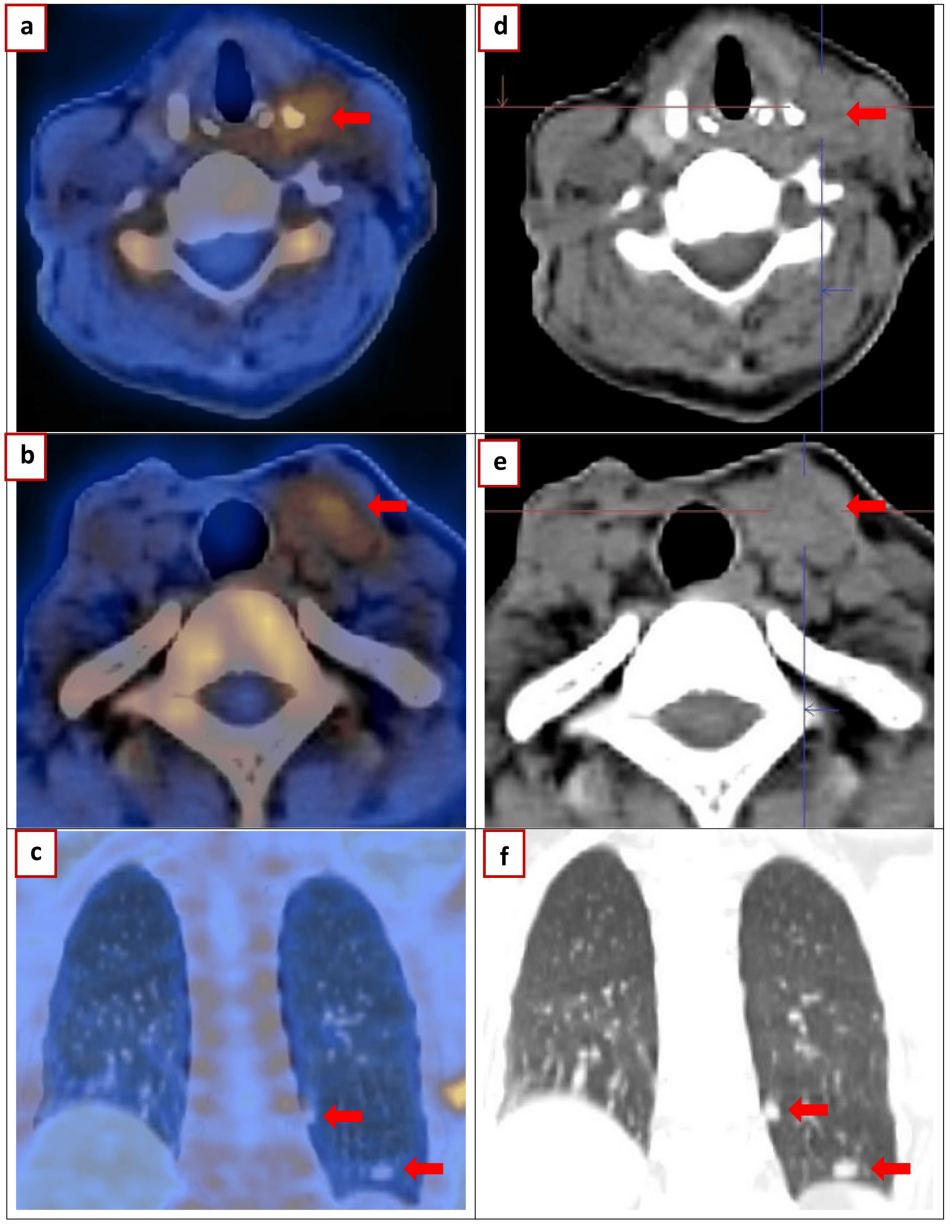
Nuclear medicine imaging (a-f) 2024 May: Gallium (Ga)-fibroblast activation protein inhibitor (FAPI) PET/CT (fibroblast activating protein; Oncoview). Ga-FAPI positron emission computed tomography imaging demonstrated high expression of fibroblast activation protein (FAP) in all tumor sites. Soft tissue nodules in the left thyroid bed and adjacent mid jugular region standardized uptake value (SUV): 1.7 (largest 15 × 11 mm; progression); left lower cervical and supraclavicular lymph nodes (largest 6 × 5 mm; regression); left lung nodules SUV: 0.8 (9 mm; additional bilateral nodules up to 9 mm; progression).

**Figure 5:**
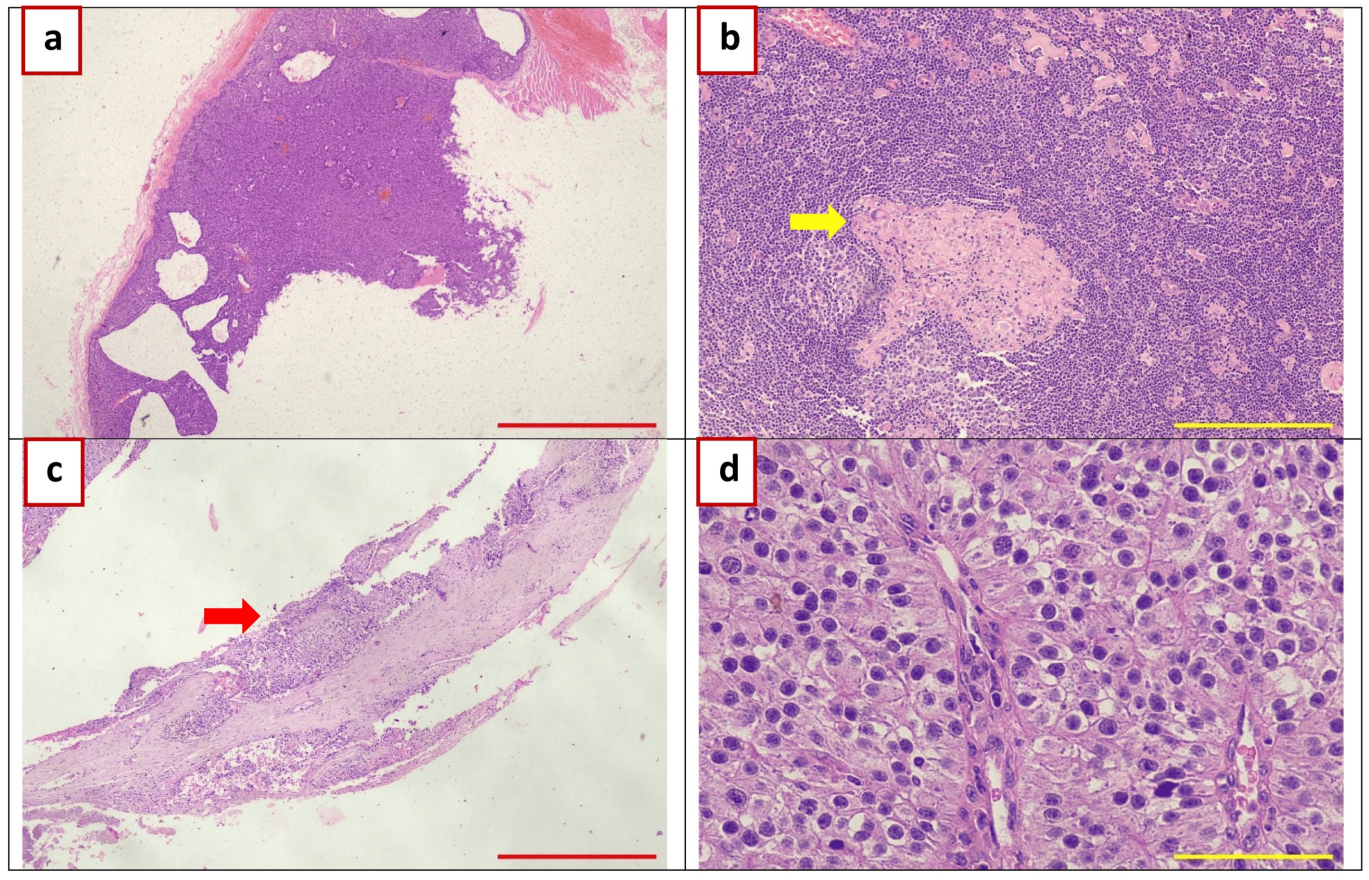
Histopathology photomicrographs 2022 January: Lymph node excision: (a) Lymph node: ×20 (scale bar = 1,000 µm). (b) Lymph node: ×100 (scale bar = 200 µm); non-caseating granulomatous lymphadenitis (possible sarcoidosis). 2022 February: Left parathyroidectomy: (c) Parathyroid carcinoma with capsular invasion; ×40 (scale bar = 500 µm). (d) Parathyroid carcinoma with oxyphilic cells; ×400 (scale bar = 50 µm). No lymph node metastasis.

In January 2022, a 41-year-old woman presented with a six-month history of progressive weakness in her lower limbs, which left her unable to walk or rise from a sitting position. She also reported difficulty holding objects, hip and back pain, and significant weight loss of 13 kg. Initially treated by orthopedic and surgical teams with analgesics, her routine laboratory tests revealed hypercalcemia, with a serum calcium level of 15.5 mg/dL (3.87 mmol/L) (reference: 8.8-10.6 mg/dL (2.20-2.65 mmol/L)) (Figure 1). Further examination identified left cervical and supraclavicular lymphadenopathy, and a subsequent biopsy revealed non-caseating granulomatous lymphadenitis. Additional tests showed a serum angiotensin-converting enzyme (ACE) level of 27.9 U/L (normal range: 12-68 U/L), a negative Mantoux test, and chest X-ray findings of hilar lymphadenopathy suggestive of possible sarcoidosis.

Diagnostic assessment

Following this initial presentation, in February 2022, she underwent further diagnostic work-up by the surgical team that confirmed primary hyperparathyroidism, with serum intact PTH level exceeding 2,500 pg/mL (>265 pmol/L) (reference: 12-88 pg/mL (1.27-9.33 pmol/L)) (Figure 1). An ultrasound of the neck reported a well-defined hypoechoic mass with increased vascularity in the left superior parathyroid area measuring 37 × 17 mm (likely neoplastic), as well as enlarged left supraclavicular lymph nodes (largest 8 × 4 mm) with distorted architecture (likely metastatic). At this stage, no endocrinology referral or nuclear medicine work-up was undertaken by the treating surgical team.

Treatment

Subsequently, she underwent left superior parathyroidectomy and left hemithyroidectomy. Histopathological examination of the excised tissue indicated PC versus adenoma with oxyphilic cell features but no evidence of lymph node metastasis (Figure 5). Postoperatively, the patient developed progressive hypocalcemia, with serum calcium dropping to 5.9 mg/dL (1.47 mmol/L) by two weeks. She was treated with calcium and vitamin D supplementation (one tablet per day consisting of 1,250 mg calcium carbonate equivalent to elemental calcium 500 mg per day and vitamin D3 cholecalciferol 250 IU).

In June 2022, for the first time, the treating surgical team sought an endocrinology referral. The evaluation indicated severe vitamin D deficiency (25-hydroxy vitamin D 3.0 ng/mL (7.5 nmol/L); reference: >30 ng/mL (>75 nmol/L)) and elevated serum PTH (394 pg/mL (41.8 pmol/L)). In retrospect, the exceedingly high initial PTH levels were likely influenced by associated long-standing severe vitamin D deficiency. A retrospective medical records (CT/MRI) review revealed (a) pseudofractures of bilateral superior pubic rami, right iliac crest, and bilateral femoral neck; (b) marrow expansion and diffuse osteopenia in all pelvic bones and femora; (c) diffuse altered signal intensities with inhomogeneous enhancement in all vertebrae, skull bones, sternum, bilateral ribs, pelvic bones, and femora (diffuse marrow replacement disorder). Her treatment included (a) a tablet of calcium citrate malate 1,250 mg equivalent to elemental calcium 250 mg three times daily; (b) a tablet of alfacalcidol 0.25 mcg three times daily; and (c) vitamin D3 cholecalciferol 600,000 IU as one intramuscular injection.

In January 2023, the patient presented with visible swelling on the right side of her neck and experienced a hypercalcemic crisis, with serum calcium levels fluctuating from 13.2 mg/dL to 19.9 mg/dL (3.30 to 4.98 mmol/L) and serum PTH levels of 572 pg/mL (60.7 pmol/L) (Figure 2). She received immediate treatment, including intravenous fluids, calcitonin, and zoledronic acid. Imaging revealed a cystic and solid mass on the right side of her neck, prompting wide excision of the mass. Histopathological examination confirmed PC with parathyromatosis. Immunohistochemical analysis showed CD31 positivity (indicating the presence of tumor cells within blood vessels in the pseudocapsule) and a Ki-67 index of around 5%.

By September 2023, the patient suffered another hypercalcemic crisis, with serum calcium at 15.0 mg/dL (3.75 mmol/L) and serum PTH at 725 pg/mL (76.9 pmol/L). Imaging identified a new nodule in the left mid-jugular area and left lower cervical/supraclavicular lymph nodes, leading to left cervical neck dissection and lymph node excision (Figure 3). Histopathological analysis confirmed PC deposits in neck soft tissues but no lymph node metastases.

Outcome and follow-up

Since September 2023 and continuing until June 2024, the patient has experienced progressive and resistant hypercalcemia with multiple recurrent hypercalcemic crises and hospitalizations. Initial treatment with cinacalcet and zoledronic acid showed limited efficacy. However, the introduction of denosumab led to dramatic improvement, with serum calcium levels decreasing from 16.7 to 7.9 mg/dL (4.18 to 1.98 mmol/L) and PTH of 1,168 pg/mL (123.9 pmol/L). Despite this response, the patient continues to be at risk due to progressive local neck lesions and potential distant metastases in the lungs.

Gallium (Ga)-fibroblast activation protein inhibitor (FAPI) PET/CT imaging demonstrated high expression of fibroblast activation protein (FAP) in all tumor sites (Figure 4). Advanced Lutetium-177 (Lu177)-FAPI-targeted radionuclide therapy is being considered pending ethical clearance and informed consent.

As of June 2024, the patient's physical examination showed a height of 147 cm, a weight of 33.8 kg, and a blood pressure of 81/58 mm Hg. Laboratory tests revealed hemoglobin of 8.7 g/dL (5.4 mmol/L) (reference: 12.3-15.3 g/dL (7.62-9.48 mmol/L)), serum albumin of 3.1 g/dL (31 g/L) (reference: 3.4 to 5.4 g/dL (34 to 54 g/L)), serum alkaline phosphatase greater than 1,000 U/L (>16.7 μkat/L) (reference: 45-129 U/L (0.75-2.15 μkat/L)), and an estimated glomerular filtration rate (eGFR) of 101 mL/min/1.73 m². Her urine albumin-to-creatinine ratio was 87.2 µg/mg of creatinine (<30), and abdominal ultrasound revealed nephrocalcinosis, bilateral renal calculi, and myometrial calcification.

The histopathological findings related to this case are summarized in Figure 5.

Key histopathological criteria for diagnosis of PC included capsular invasion, vascular invasion and increased mitotic activity (increased mitotic figures, atypical mitoses), and cytological atypia (nuclear atypia, including irregular nuclear contours, prominent nucleoli, and increased nuclear-to-cytoplasmic ratio).

Immunohistochemical analysis showed CD31 positivity (indicating the presence of tumor cells within blood vessels in pseudocapsule) and a Ki-67 index of around 5%. Ki-67 index of >5% is often considered suggestive of malignancy, while benign parathyroid lesions typically have lower Ki-67 indices. Higher Ki-67 indices (e.g., >10%) may be associated with more aggressive behavior and poor prognosis. For other endocrine tumors in general (such as neuroendocrine tumors), the Ki-67 index is often used to grade the tumor, for example, grade 1: Ki-67 index <3%; grade 2: Ki-67 index 3-20%; and grade 3: Ki-67 index >20%. Exact thresholds can vary depending on the specific type of endocrine tumor and the grading system used.

On follow-up to date, no additional manifestations of granulomatous lymphadenitis or related pathologies have been observed; hence, no specific treatment has been initiated. Granulomatous diseases like sarcoidosis (non-caseating) can present with “non-PTH” mediated hypercalcemia.

## Discussion

PC is one of the rarest malignancies. In this presentation, we summarize the ongoing medical saga of a young lady with PC (left superior PC; parathyromatosis on the right side of the neck; neck soft tissue carcinoma deposits on the left side; lung metastases). Coincidental granulomatous lymphadenitis and nutritional vitamin D deficiency resulted in additional diagnostic and management challenges. Denosumab was successfully used in managing her refractory hypercalcemia [7-9].

Multiple nodules of hyperfunctioning parathyroid tissue forming in the neck and/or mediastinum characterize the phenomenon referred to as “parathyromatosis,” a rare cause of recurrent hyperparathyroidism. Parathyromatosis most commonly occurs as a secondary phenomenon following neck surgery (intraoperative seeding) for hyperparathyroidism due to suboptimal handling of glands during neck exploration. Fine needle aspiration is not recommended because of the risk of parathyromatosis [10-12]. Histopathologically, parathyromatosis is distinguished by the presence of multiple small, non-encapsulated nodules of hypercellular parathyroid tissue, often consisting of chief cells, oxyphil cells, and transitional cells. These nodules are typically 1-2 mm in size and scattered within soft tissues, making detection through imaging challenging. The condition may rarely develop spontaneously, possibly due to aberrant embryological development [13].

PC poses significant diagnostic challenges due to its rarity and the often-ambiguous nature of its histopathological features. Differentiating between benign parathyroid adenomas and malignant carcinomas can be difficult, as both can present with similar histological characteristics, such as oxyphilic cell populations and a lack of definitive capsular invasion in initial biopsies. Diagnosis often necessitates reliance on clinical and biochemical markers, such as significantly elevated serum calcium and PTH levels, and the presence of a palpable neck mass. However, definitive diagnosis frequently occurs only after local recurrence or metastasis becomes evident. A Ki-67 index of >5% is often considered suggestive of malignancy, while benign parathyroid lesions typically have lower Ki-67 indices. Higher Ki-67 indices (e.g., >10%) may be associated with more aggressive behavior and poor prognosis. Other applications of immunohistochemistry in parathyroid tumors include analysis of loss of parafibromin and other features of an abnormal immunophenotype, hinting towards carcinoma [14].

Surgical resection with negative margins is the cornerstone of managing PC, but achieving clear margins can be particularly challenging due to the tumor’s infiltrative nature and its proximity to critical neck structures [15]. In our case, incomplete resection during initial surgery necessitated multiple subsequent interventions, underscoring the aggressive nature of PC and the difficulty of achieving complete tumor removal. Extensive surgery increases the risk of damage to the thyroid, trachea, or esophagus, making precise preoperative imaging crucial for surgical planning. Recurrent laryngeal nerve injury is a common complication that can lead to vocal cord paralysis and hoarseness; it can be mitigated by careful identification of the nerve and the use of intraoperative nerve monitoring. Postoperative hypocalcemia is a common complication due to the abrupt drop in PTH levels and pre-existing bone disease from long-standing hyperparathyroidism. To prevent hungry bone syndrome after parathyroid surgery, preoperative correction of vitamin D deficiency is essential. Postoperatively, aggressive oral calcium and calcitriol are recommended to prevent hypocalcemia due to rapid bone remineralization [16]. In our patient, severe nutritional vitamin D deficiency exacerbated the risk of postoperative hypocalcemia and complicated management.

Recurrences are a common issue in PC, with rates as high as 30-70% reported in the literature [17]. Complete en-bloc resection with clear margins minimizes recurrence. Reoperation is often required for resectable recurrent disease, but each surgery carries increased risks and potential complications. For unresectable or metastatic disease, medical management of hypercalcemia becomes crucial. Adjuvant therapy with chemotherapy or external beam radiation has not been proven to affect disease-free or overall survival [18-19].

Managing recurrent and resistant hypercalcemia in PC patients is challenging. Our patient continues to require multiple pharmacological interventions, including calcitonin, zoledronic acid, and cinacalcet, with the best response observed with denosumab. Denosumab, a monoclonal antibody against RANKL, has shown efficacy in controlling hypercalcemia of malignancy and could be a valuable addition to therapy in refractory cases. Cinacalcet, a calcimimetic agent, is indicated to treat hypercalcemia in PC and is particularly beneficial in cases of recurrent or inoperable PC [20]. Hydration (intravenous saline in acute settings), loop diuretics (furosemide), and dietary modifications (avoiding calcium-rich foods like dairy products) should be implemented as per guidelines.

The prognosis for PC is highly variable, with 5- and 10-year survival rates between 75-100% and 50-90%, respectively. Key factors influencing prognosis include the completeness of initial surgical resection, the presence of metastases, and the ability to manage hypercalcemia effectively. In this patient, the recurrent and progressive nature of hypercalcemia, despite aggressive management, underscores the challenges in achieving long-term disease control. Ultimately, patients succumb to the sequelae of hypercalcemia rather than tumor burden [3].

## Conclusions

PC is one of the rarest malignancies. The critical importance of achieving complete surgical resection with negative margins during the initial treatment of PC has been highlighted, as this significantly reduces recurrence rates and improves patient prognosis. The necessity for histopathological vigilance cannot be overstated, as the potential for ambiguity in histological findings demands thorough and repeated evaluations to confirm malignancy accurately. Furthermore, the presence of coexisting conditions such as granulomatous lymphadenitis and severe vitamin D deficiency complicates the clinical picture, highlighting the need for a comprehensive and multidisciplinary approach to patient management.

The persistent challenge of recurrent hypercalcemia in PC necessitates a multifaceted pharmacological strategy, with therapies like denosumab showing promise in managing refractory cases. As we look to the future, novel therapies, including FAPI-targeted radiotherapy, represent potential advancements in the treatment of advanced or metastatic PC. These innovations emphasize the need for ongoing research and clinical trials to improve outcomes and refine treatment strategies for this rare and complex malignancy.

## Appendices

Appendix A 

**Table 2:** Serial radiological and nuclear medicine imaging observations

Year and month	Radiological and nuclear medicine imaging observations
2022 January	Ultrasound neck: Well-defined hypoechoic mass with increased vascularity left superior parathyroid area 37 × 17 mm (likely neoplastic). Enlarged left supraclavicular lymph nodes (largest 8 × 4 mm) with distorted architecture (likely metastatic).
2022 January	Ultrasound abdomen pelvis: Normal.
2022 January	Bone mineral density DEXA: T scores: L1-L4 -5.5; femur neck right: -4.9; femur neck left: -4.7; forearm radius distal: -7.2.
2022 January	CT scan hip joints: Pseudofractures bilateral superior pubic rami and right iliac crest and left femoral neck. Marrow expansion and diffuse osteopenia all pelvic bones and femora.
2022 January	MRI whole spine: Diffuse altered signal intensities with inhomogeneous enhancement in all vertebrate, skull bones, sternum, bilateral ribs, pelvic bones and femora. diffuse marrow replacement disorder.
2022 June	CT scan hip joints: Pseudofractures bilateral superior pubic rami and right iliac crest. Progression of fracture of left femoral neck. New fracture right femoral neck. Marrow expansion and diffuse osteopenia all pelvic bones and femora.
2022 June	C11-choline PET-CT: Soft tissue (13 × 11 mm) in the left thyroid bed SUV: I.9 - bilateral lower cervical/supraclavicular lymph nodes SUV: 2
2022 June	Ga-68 DOTANOC PET-CT: No abnormal Ga-68 DOTANOC accumulation.
2023 January	Ultrasound neck: Small round to oval hypoechoic lesion 17 × 8 mm in the subcutaneous plane of right paramedian strap muscles. Metastatic lymph node?
2023 January	99m Tc sestamibi SPECT CT scan: Subcutaneous cystic lesion anterior to right lobe of the thyroid. No other evidence of abnormal focal increased MIBI uptake in neck or mediastinum.
2023 January	C11-choline PET-CT: Subcutaneous nodule is noted in the right lower anterior neck SUV: 5.8 (17 × 15 mm). Left lower cervical and supraclavicular lymph nodes SUV: 2.2 (largest 11 × 9 mm). Left lung nodule (5.5 mm).
2023 March	Tc99 MDP isotope bone scan: Diffuse increased osteoblastic activity of entire skeleton - metabolic bone disease. No evidence of focal lesions suggestive of fractures or metastases.
2023 August	FDG-PET-CT: - “New” nodule / lymph node in the left mid jugular region SUV: 4.8 (8 × 6 mm). Left lower cervical and supraclavicular lymph nodes SUV: 1.9 (largest 11 × 9 mm). Left lung nodules SUV: 1.6 (7.5 mm; two additional nodules up to 6 mm; progression).
2023 August	Ga-68 DOTANOC PET-CT: “New” nodule / lymph node in the left mid jugular region SUV: 3.2 (8 × 6 mm). Left lower cervical and supraclavicular lymph nodes SUV: 1.6 (largest 11 × 9 mm). Left lung nodules SUV: 1.6 (7.5 mm; two additional nodules up to 6 mm; progression).
2024 May	Ga-FAPI PET-CT (fibroblast activating protein; Oncoview). Soft tissue nodules in the left thyroid bed and adjacent mid jugular region SUV: 1.7 (largest 15 × 11 mm; progression). Left lower cervical and supraclavicular lymph nodes (largest 6 × 5 mm; regression). Left lung nodules SUV: 0.8 (9 mm; additional bilateral nodules up to 9 mm; progression). Diffuse uptake is noted in the skeletal lesions. Non-specific uptake is seen in the pancreas, likely inflammatory versus infectious etiology.
2024 May	PET-CT summary: Status post left hemi-thyroidectomy and excision of the parathyroid lesion. Status post excision of new subcutaneous nodule in the right lower anterior neck. Interval increase in soft tissue nodules in the left thyroid bed and adjacent mid jugular region - likely recurrence (largest one now measuring 15 × 11 mm, previously measuring 8 × 6 mm). Interval regression of small left lower cervical and supraclavicular lymph nodes (largest now measuring 6 × 5 mm, previously measuring 11 × 9 mm). Further interval increases in size and number of metastatic left lower lobe lung nodules (now measuring up to 9 mm, previously 7.5 mm). There is interval development of additional small bilateral pulmonary nodules (measuring up to 9 mm). Redemonstration of heterogeneous skeletal sclerosis. Interval development of peripancreatic fat stranding and thin fluid - pancreatitis.
2024 June	Ultrasound abdomen pelvis: Nephrocalcinosis, bilateral renal calculi, myometrial calcification.

Appendix B 

**Table 3:** Chronological summary of the clinical, imaging, surgical, and histopathology details

Year and month	Clinical features	Imaging	Surgery	Histopathology
2022 January	Hypercalcemia, weight loss and proximal muscle weakness	Left cervical and supraclavicular lymphadenopathy	Lymph node excision	Non-caseating granulomatous lymphadenitis (possible sarcoidosis).
2022 February	Primary hyperparathyroidism	Left parathyroid adenoma	Left parathyroid adenomectomy, left hemithyroidectomy	Parathyroid carcinoma versus adenoma with oxyphilic cells, no lymph node metastasis.
2023 January	New swelling on right side of neck and hypercalcemic crisis	Right cystic and solid neck mass	Wide excision of the right-sided neck mass	Low-grade parathyroid carcinoma. Immunohistochemistry CD31 positivity (presence of tumor cells within blood vessels in the pseudocapsule), and Ki-67 extremely low (5%).
2023 September	Hypercalcemic crisis	New nodule in the left mid-jugular area and left lower cervical/supraclavicular lymph nodes.	Left cervical neck dissection and lymph node excision	Parathyroid carcinoma deposits in neck soft tissues. No lymph node metastases.
